# RETRACTION: Effect of Quality Nursing Care on Wound Pain and Anxiety in Burn Patients: A Meta‐Analysis

**DOI:** 10.1111/iwj.70450

**Published:** 2025-03-26

## Abstract

**RETRACTION:** YangL. , YuanB.‐Q. , JuY.‐Y. , LiuW. , WangY.‐P. , “Effect of Quality Nursing Care on Wound Pain and Anxiety in Burn Patients: A Meta‐Analysis,” International Wound Journal 21, no. 4 (2024): e14798, 10.1111/iwj.14798.38572761 PMC10993347

The above article, published online on 04 April 2024, in Wiley Online Library (http://onlinelibrary.wiley.com/), has been retracted by agreement between the journal Editor in Chief, Professor Keith Harding; and John Wiley & Sons Ltd. Following an investigation by the publisher, all parties have concluded that this article was accepted solely on the basis of a compromised peer review process. The editors have therefore decided to retract the article. The authors did not respond to our notice regarding the retraction.

**RETRACTION:** L. Yang , B.‐Q. Yuan , Y.‐Y. Ju , W. Liu , Y.‐P. Wang , “Effect of Quality Nursing Care on Wound Pain and Anxiety in Burn Patients: A Meta‐Analysis,” International Wound Journal 21, no. 4 (2024): e14798, 10.1111/iwj.14798.38572761 PMC10993347

The above article, published online on 04 April 2024, in Wiley Online Library (http://onlinelibrary.wiley.com/), has been retracted by agreement between the journal Editor in Chief, Professor Keith Harding; and John Wiley & Sons Ltd. Following an investigation by the publisher, all parties have concluded that this article was accepted solely on the basis of a compromised peer review process. The editors have therefore decided to retract the article. The authors did not respond to our notice regarding the retraction.

